# Associations Between Sleep Quality, Mental Health, and Vitality in Professional Drivers: A Cross-Sectional Study

**DOI:** 10.3390/clockssleep8030053

**Published:** 2026-08-29

**Authors:** Gabriela Roxana Louisse Neacşu, Agripina Rașcu

**Affiliations:** 1 Doctoral School, Carol Davila University of Medicine and Pharmacy, 020021 Bucharest, Romania; 2 Clinical Department 5, Faculty of General Medicine, Carol Davila University of Medicine and Pharmacy, 020021 Bucharest, Romania; 3 Department of Occupational Medicine, Colentina Clinical Hospital, 020125 Bucharest, Romania

**Keywords:** sleep quality, mental health, vitality, health-related quality of life, professional drivers, occupational health

## Abstract

Professional drivers are exposed to work conditions that may affect sleep quality. Although poor sleep quality has been associated with lower mental health (MH) and vitality (VT), its relationship with these outcomes in professional drivers remains unclear. This cross-sectional study examined the association between sleep quality and selected health-related quality-of-life (HRQoL) domains among 399 professional drivers. Sleep quality was assessed using the Pittsburgh Sleep Quality Index (PSQI). At the same time, HRQoL was evaluated using the Mental Health, Vitality, and Bodily Pain domains of the 36-Item Short Form Health Survey. Correlation and regression analyses were performed. The mean PSQI score was 8.59, indicating overall poor sleep quality. Higher PSQI scores were negatively associated with MH (ρ = −0.384, *p* < 0.001) and VT (ρ = −0.495, *p* < 0.001). Sleep quality remained an independent predictor of MH (B = −2.47, *p* < 0.001; R^2^ = 0.155) and VT (B = −4.10, *p* < 0.001; R^2^ = 0.268), whereas age showed a modest association only with VT (B = −0.235, *p* = 0.035). Poor sleep quality was associated with lower MH and VT scores among professional drivers. The stronger association observed for VT suggests that sleep quality may be particularly relevant to energy levels and daily functioning in this study population.

## 1. Introduction

Urban public transport operators work under demanding conditions that require sustained attention, rapid decision-making, and continuous responsibility for passenger safety. Adequate sleep is essential for maintaining these abilities, as sleepiness and reduced vigilance impair attention, concentration, reaction time, and other driving-related cognitive functions [1].

Poor sleep quality has also been associated with reduced occupational well-being [2] and lower health-related quality of life (HRQoL) across multiple physical and psychological domains [3].

A recent systematic review reported a consistent association between occupational stress and poor sleep quality across various occupational groups [4].

HRQoL reflects the perceived impact of health on physical, psychological, and social functioning. Among its dimensions, Mental Health (MH) reflects psychological well-being, whereas Vitality (VT) reflects energy levels and fatigue. Previous studies have shown that impaired sleep quality and poor sleep hygiene are associated with lower MH and VT scores, particularly among shift workers [5].

Although the relationship between sleep quality and HRQoL has been widely investigated, evidence specifically examining the MH and VT domains in professional drivers remains limited. In particular, it is unclear whether sleep quality is associated with these HRQoL dimensions independently of demographic and occupational characteristics.

Therefore, the present study examined the relationship between sleep quality and the MH and VT domains of HRQoL in a study population of professional drivers. MH and VT were selected as the predefined outcome measures because they represent the psychological and energy-related dimensions of HRQoL that are most closely linked to sleep quality. Particular attention was given to whether sleep quality remained associated with these outcomes after accounting for selected demographic and occupational characteristics.

## 2. Results

### 2.1. Sample Characteristics

The study population consisted of 399 professional drivers, with an average age of 51.2 years (SD = 5.76) and an average career longevity of 29.8 years (SD = 7.39), reflecting a predominantly experienced workforce. The sample was predominantly male (95.2%), with females accounting for just 4.8%. The majority were current smokers (60.4%), followed by non-smokers (26.1%) and former smokers (13.5%). In terms of work schedules, 60.1% worked day shifts, 21.3% worked rotating shifts and 18.5% worked night shifts (see Table 1).

### 2.2. Descriptive Analysis of Clinical Variables

PSQI scores ranged from 6 to 16, with a mean value of 8.59 (SD = 1.83), indicating generally poor sleep quality across the study population. HRQoL scores were relatively high, with mean values of 87.9 (SD = 11.9) for MH and 81.3 (SD = 14.9) for VT. The BP domain, analysed as a secondary outcome, had a mean score of 76.7 (SD = 23.8) (Table 2).

Descriptive statistics for the remaining SF-36 domains are presented in Supplementary Table S1.

### 2.3. Correlational Analysis

Sleep quality was significantly associated with all examined domains of HRQoL (VT, MH and BP). Higher PSQI scores were moderately correlated with lower VT (ρ = −0.495, *p* < 0.001) and lower MH (ρ = −0.384, *p* < 0.001). Additionally, BP was significantly correlated with both VT and MH. All correlations are presented in Table 3.

To further characterise health-related quality of life, the SF-36 Physical Component Summary (PCS) and Mental Component Summary (MCS) scores were also calculated according to the standard SF-36 scoring methodology [6]. PCS and MCS were expressed as norm-based summary scores (mean = 50, SD = 10 in the reference population) and are therefore not directly comparable with the individual SF-36 domain scores reported on the 0–100 scale. Descriptive statistics are presented in Supplementary Table S2, correlations with the individual SF-36 domains in Supplementary Table S3, and correlations with the PSQI global score in Supplementary Table S4.

### 2.4. Differences Across Work Schedules

Table 4 summarises the comparison of sleep quality, mental health, vitality, and BMI across work schedules. No statistically significant differences were observed in MH (*p* = 0.441), VT (*p* = 0.299), or BMI (*p* = 0.158) across work schedules. Although sleep quality showed a non-significant trend towards poorer outcomes among rotating shift workers (*p* = 0.054), pairwise comparisons did not reveal any significant effects.

### 2.5. Predictors of Mental Health and Vitality

In the linear regression analysis, sleep quality was found to have a significant independent association with MH, explaining 15.5% of the variance (R^2^ = 0.155). In contrast, age did not reach statistical significance. A second model demonstrated a similarly strong association between sleep quality and VT, accounting for 26.8% of the variance (R^2^ = 0.268). In contrast to MH, however, age was significantly associated with lower vitality scores.

**Figure 1:**
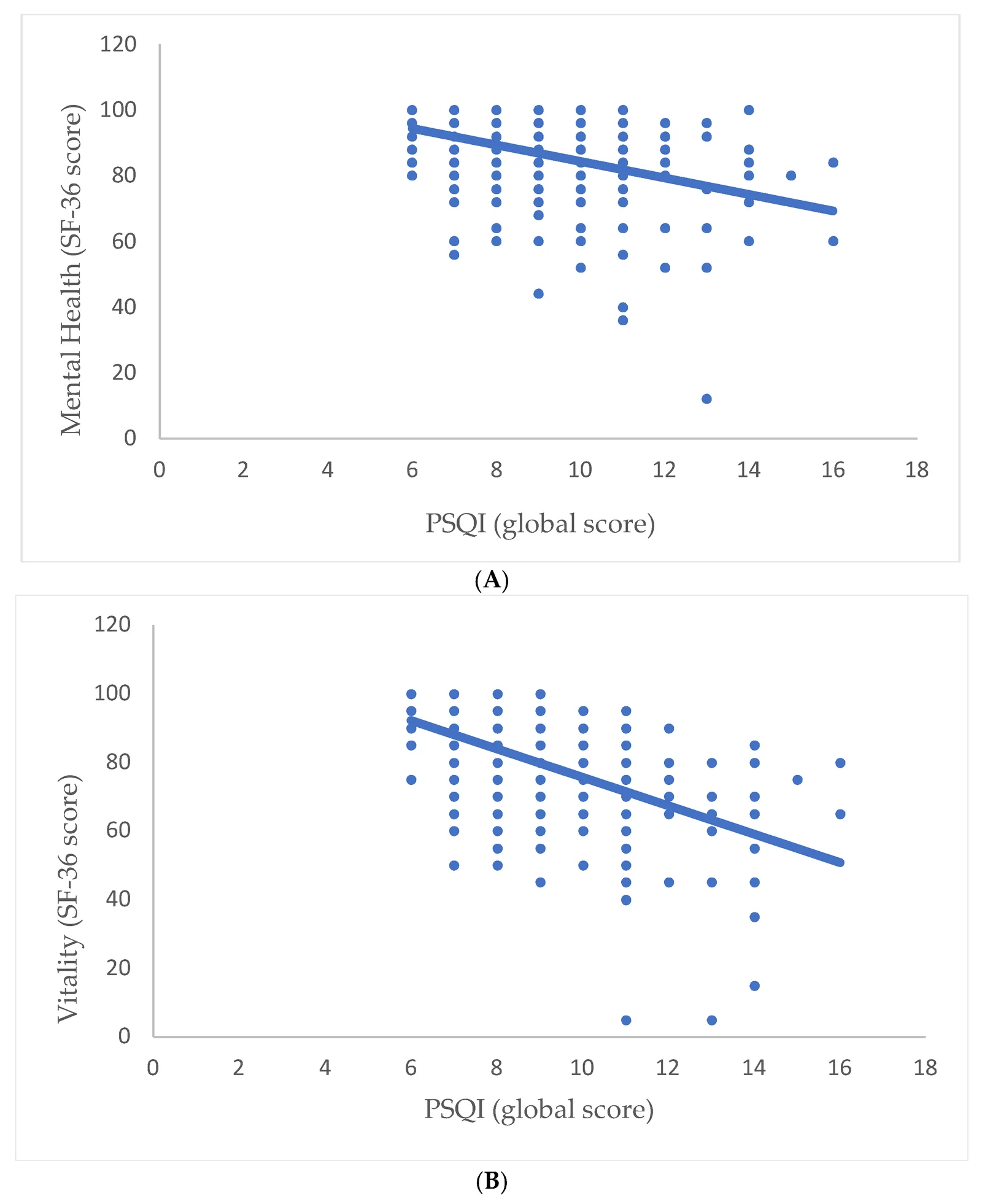
(**A**). Association between sleep quality (PSQI) and Mental Health (SF-36 score). (**B**). Association between sleep quality (PSQI) and Vitality (SF-36 score).

The detailed results of the regression models are presented in Table 5, and the relationship between sleep quality and the two HRQoL domains is illustrated in Figure 1A,B.

To further evaluate the independent contribution of sleep quality beyond demographic and occupational characteristics, we performed hierarchical regression analyses (Table 6).

In this study, hierarchical regression analyses demonstrated that demographic and occupational variables explained only a small proportion of the variance in both MH (R^2^ = 0.022) and VT (R^2^ = 0.027). After the addition of PSQI scores, the explained variance increased significantly for both MH (ΔR^2^ = 0.140, *p* < 0.001) and VT (ΔR^2^ = 0.245, *p* < 0.001), indicating that sleep quality contributed substantial independent explanatory value beyond demographic and occupational characteristics.

## 3. Discussion

In this population of urban public transport drivers, sleep quality was consistently associated with both MH and VT. These associations remained evident after adjustment for age, suggesting that current sleep status may be more closely related to wellbeing than demographic characteristics alone.

Although the overall HRQoL scores were relatively high, poorer sleep quality was associated with lower scores in the Mental Health and Vitality domains.

### 3.1. The Importance of Sleep over Demographic Factors

Sleep quality was more strongly associated with both MH and VT than age. In the regression models, PSQI remained independently associated with both outcomes, while age had only a modest, domain-specific effect, reaching statistical significance only for VT.

Similar links have been reported in occupational populations, showing that sleep-related factors may be more closely associated with psychological wellbeing than demographic characteristics [7].

This pattern suggests that sleep quality may be more relevant to psychological wellbeing than accumulated occupational experience, a finding further supported by the hierarchical regression models, in which PSQI explained additional variance beyond demographic and occupational characteristics.

### 3.2. Sleep Quality and Health-Related Quality of Life

Among the drivers included in this study, poorer sleep quality was associated with lower scores in both MH and VT domains. Similar associations have been reported previously in occupational populations [7]. The correlation was stronger for VT than MH, suggesting that energy depletion and reduced vitality may represent functional mechanisms linking sleep disturbances to psychological wellbeing.

Previous studies have also linked sleep disturbances and daytime sleepiness with fatigue and reduced functional capacity. In this context, VT may be understood as the functional expression of fatigue-related processes, reflecting diminished energy levels and the ability to sustain daily activities [8].

Comparable findings have been described in shift-working populations, where poorer sleep quality has been associated with lower quality-of-life scores [5]. Evidence from other populations has similarly shown that poor sleep quality is associated with lower scores across multiple SF-36 domains, particularly VT and MH [9].

Longitudinal evidence has shown that poorer sleep quality is associated with less favourable HRQoL trajectories over time. These observations are in line with our findings, where sleep quality showed a stronger association with VT than with demographic characteristics [10,11].

### 3.3. Bodily Pain as a Secondary Finding

Although Bodily Pain was not a primary outcome of this study, lower BP scores were associated with poorer sleep quality and lower Mental Health scores. As the SF-36 assesses pain in general terms, without distinguishing its type, duration or location, these findings should be interpreted with caution. Previous studies have reported a close relationship between sleep disturbances, pain and mental health, with each of these factors potentially influencing the others [12].

Some evidence also indicates that sleep disturbances may be a stronger predictor of future pain than pain is of subsequent sleep impairment [13]. While our findings are consistent with this observation, the present study was not designed to investigate pain characteristics in professional drivers. Further studies including detailed measures of musculoskeletal symptoms or chronic pain are needed to understand this relationship better.

### 3.4. Work Schedules and the Absence of Group Differences

Contrary to expectations, no statistically significant differences were observed across work schedules in terms of MH, VT, or BMI. Although a trend toward poorer sleep quality was observed among rotating shift workers, this did not reach statistical significance. This finding is consistent with previous studies reporting no meaningful differences in burnout and psychological distress between shift and non-shift workers in large occupational cohorts [14].

The available evidence remains mixed, with some studies reporting poorer sleep quality and quality of life among shift workers, while others have found non-significant or heterogeneous associations depending on the population studied [15,16].

In this context, the absence of significant differences observed in the present study may indicate that individual variability in sleep quality outweighs the effect of the work schedule alone. Individual coping strategies or workplace conditions may partially explain the absence of significant differences between work schedules.

Additionally, the relatively homogeneous nature of the sample, consisting exclusively of professional drivers, may have reduced between-group variability and limited the detection of schedule-related differences.

### 3.5. Implications for Occupational Health

The findings may also be relevant in occupational health practice. In this population, sleep quality was associated with both MH and VT, suggesting that sleep-related complaints may provide useful information about worker wellbeing. Validated questionnaires such as the PSQI can be used to identify workers reporting poor sleep quality and related functional difficulties [17,18].

Measures targeting sleep hygiene, recovery opportunities, and physical discomfort may offer practical opportunities for improving sleep and perceived wellbeing. These strategies could be integrated into routine occupational health assessments, contributing to early identification and targeted prevention of functional impairment.

Given the nature of professional driving, maintaining optimal vitality levels is particularly important, as reduced energy and alertness may directly affect operational safety. This functional relevance is reinforced by evidence showing that sleepiness and reduced vigilance impair attention, concentration, and reaction time, which are essential for safe driving performance [1].

### 3.6. Limitations and Future Directions

This cross-sectional study has several limitations that should be considered. First, the cross-sectional design does not permit causal inference about the relationship between sleep quality and HRQoL.

Second, all measures were based on self-reported questionnaires, which may introduce reporting bias, particularly in occupational groups where symptom underreporting may occur.

Although objective sleep assessments were available for a subset of participants, they were not available for the entire study population and were therefore not included in the present analysis, which focused on subjective measures.

Third, the relatively homogeneous sample limits the generalizability of the findings to other occupational groups. In addition, the group was predominantly male, which may further restrict the applicability of the results to more diverse populations. Selection bias should also be considered, as individuals who choose a professional driving career may differ from the general population in terms of physical fitness, psychological characteristics, coping strategies, or occupational demands. The relatively narrow range of PSQI scores should also be considered when interpreting the findings, as limited variability may have attenuated the observed associations.

Future longitudinal studies are needed to determine whether improvements in sleep quality are associated with measurable changes in VT and MH over time. In addition, integrating both subjective and objective sleep assessments (e.g., actigraphy or polygraphy) would strengthen the validity of the observed associations and provide a more comprehensive evaluation of sleep-related outcomes.

## 4. Materials and Methods

### 4.1. Study Design and Participants

This study employed a cross-sectional analytical design. The study population consisted of 399 professional drivers operating within an urban public transport system. Participants were consecutively recruited during their mandatory periodic medical and psychological evaluations between October 2023 and December 2025. Inclusion criteria required that participants be active employees at the time of the study and provide informed consent. Exclusion criteria were incomplete questionnaire data or withdrawal of consent. All eligible individuals who were invited agreed to participate, resulting in a participation rate of 100%.

### 4.2. Measures and Instruments

Sleep quality was assessed using the Pittsburgh Sleep Quality Index (PSQI), a validated self-reported questionnaire that evaluates sleep patterns over the previous month. The global PSQI score ranges from 0 to 21, with higher scores indicating poorer sleep quality. Although the PSQI score was analysed as a continuous variable in all statistical analyses, a global score > 5 is generally considered indicative of poor sleep quality [17]. HRQoL was measured using the 36-Item Short Form Health Survey [19].

Three SF-36 domains were included in the present analysis: MH, VT and Bodily Pain (BP). Each domain was transformed into a scale of 0–100, according to the standard SF-36 scoring procedure recommended by the questionnaire developers, where higher values indicate better perceived health and functional status. MH and VT were selected as the primary outcomes because of their relevance to psychological wellbeing and perceived energy levels, while BP was analysed as a secondary outcome.

### 4.3. Demographic and Occupational Variables

Additional data were collected on sex, work schedule, total career longevity, smoking status, and body mass index (BMI). Although sex was recorded as a demographic variable, it was not included in the inferential analysis due to its highly imbalanced distribution in the sample. Work schedules were categorised as fixed day shifts, fixed night shifts or rotating shifts. Total career longevity was defined as the cumulative number of years spent in the workforce. Smoking status was classified as non-smoker, current smoker, or former smoker (defined as individuals who had quit smoking at least six months before the study). BMI was calculated based on self-reported height and weight.

### 4.4. Statistical Analysis

Statistical analyses were performed using Jamovi software (version 2.6) [20]. Descriptive statistics were used to summarise the demographic and clinical characteristics. One-way analysis of variance (ANOVA) with Welch correction and Games–Howell multiple comparison tests were used to assess differences across work schedules.

Associations between sleep quality and HRQoL domains were evaluated using Spearman’s rank correlation coefficients. Multiple linear regression models were constructed to examine the independent effect of sleep quality on MH and VT, adjusting for age. Additional hierarchical regression analyses were subsequently performed to evaluate the contribution of sleep quality beyond demographic and occupational variables. Regression assumptions were assessed by visual inspection of residual Q-Q plots and residuals-versus-fitted plots. Given some departures from residual normality and homoscedasticity, sensitivity analyses using HC3 heteroscedasticity-robust standard errors were performed; the main findings regarding PSQI remained unchanged. A two-sided *p*-value of <0.05 was considered statistically significant.

## 5. Conclusions

Poorer sleep quality was associated with lower MH and VT scores among professional drivers. The association was stronger for VT, suggesting that reduced energy and fatigue may represent important pathways linking sleep disturbances to overall wellbeing. These associations remained significant after accounting for demographic and occupational characteristics. Given the importance of alertness and functional capacity in professional driving, sleep quality deserves attention as part of a broader assessment of worker health. Further studies using longitudinal designs and objective sleep measures are needed to understand these relationships better.

## Figures and Tables

**Table 1:** Demographic and occupational characteristics of the study population (*N* = 399).

Variable	Value
Age (years)	51.2 ± 5.76
Age range	28–63
Career longevity (years)	29.8 ± 7.39
Career longevity range	4–42
Smoking status, *n* (%)	
Current smokers	241 (60.4%)
Non-smokers	104 (26.1%)
Former smokers	54 (13.5%)
Work schedule, *n* (%)	
Day shift	240 (60.1%)
Night shift	74 (18.5%)
Rotating shift	85 (21.3%)

Values are presented as mean ± standard deviation or number (percentage).

**Table 2:** Descriptive statistics of sleep quality and HRQoL measures.

Variable	Mean ± SD	Min–Max
PSQI (global score)	8.59 ± 1.83	6–16
Mental Health	87.9 ± 11.9	12–100
Vitality	81.3 ± 14.9	5–100
Bodily Pain	76.7 ± 23.8	0–100

**Table 3:** Spearman correlation between sleep quality and HRQoL Domains.

	1	2	3	4
1. Vitality	-			
2. Mental Health	0.533 ***	-		
3. PSQI (global score)	−0.495 ***	−0.384 ***	-	
4. Bodily Pain	0.442 ***	0.312 ***	−0.358 ***	-

Note: *** *p* < 0.001.

**Table 4:** Comparison of PSQI global score, MH, VT and BMI across work schedules.

Variable	Day Shift (*n* = 240)	Night Shift (*n* = 74)	Rotating Shift (*n* = 85)	*p*-Value
PSQI	8.52 ± 1.77	8.32 ± 1.73	9.02 ± 2.02	0.054
MH	88.20 ± 10.46	88.54 ± 15.39	86.35 ± 12.38	0.441
VT	81.81 ± 13.02	82.50 ± 17.31	78.82 ± 17.16	0.299
BMI	31.66 ± 5.24	30.38 ± 5.73	32.02 ± 5.73	0.158

Values are presented as mean ± standard deviation.

**Table 5:** Linear regression models predicting Mental Health and Vitality.

Outcome	Predictor	B (Estimate)	Standardized β	*p*-Value	R^2^
Mental Health	PSQI	−2.47	−0.379	<0.001	0.155
	Age	−0.173	−0.084	0.071	
Vitality	PSQI	−4.10	−0.504	<0.001	0.268
	Age	−0.235	−0.091	0.035	

**Table 6:** Hierarchical regression models examining the independent associations of sleep quality with Mental Health and Vitality.

Outcome	Model	Predictors	R^2^	ΔR^2^	F Change	*p*
Mental Health	1	Age, BMI, career longevity, work schedule	0.022	−	−	−
	2	Model 1 + PSQI	0.162	0.140	65.6	<0.001
Vitality	1	Age, BMI, career longevity, work schedule	0.027	−	−	−
	2	Model 1 + PSQI	0.271	0.245	132.0	<0.001

## Data Availability

The data presented in this study are available upon reasonable request from the corresponding authors due to privacy and ethical restrictions.

## Supplementary Materials

The following supporting information can be downloaded at https://www.mdpi.com/article/10.3390/clockssleep8030053/s1, Table S1. Descriptive statistics of SF-36 domains in the study population. Table S2. Descriptive statistics of the SF-36 Physical Component Summary (PCS) and Mental Component Summary (MCS) scores. Table S3. Correlations between SF-36 domain scores and the Physical Component Summary (PCS) and Mental Component Summary (MCS) scores. Table S4. Spearman correlations between the Pittsburgh Sleep Quality Index (PSQI) global score and the SF-36 summary component scores.
