# Supplementary material for: Associations Between Sleep Quality, Mental Health, and Vitality in Professional Drivers: A Cross-Sectional Study
Source: Clocks Sleep. 2026 Aug 29;8(3):53. doi: 10.3390/clockssleep8030053 (PMC13605273; doi:10.3390/clockssleep8030053)
Supplement: Supplementary file 1 [file clockssleep-08-00053-s001.zip › clockssleep-4445086-supplementary.pdf]

Supplementary **Table S1**. Descriptive statistics of SF-36 domains in the study population

	Physical Functioning	Role Physical	Bodily Pain	General Health	Vitality	Social Functioning	Role Emotional	Mental Health
Mean	80.6	87.3	76.7	75.0	81.3	91.7	94.3	87.9
Standard Deviation	22.7	27.7	23.8	16.9	14.9	14.2	20.5	11.9
Minimum	0	0	0	10	5	12.5	0	12
Maximum	100	100	100	100	100	100	100	100

Supplementary **Table S2**. Descriptive statistics of the SF-36 Physical Component Summary (PCS) and Mental Component Summary (MCS).

Variable	Mean ± SD
PCS	48.3 ± 8.4
MCS	58.6 ± 5.9

Note: PCS and MCS are expressed as norm-based scores (mean = 50, SD = 10 in the reference population) and are not directly comparable with the individual SF-36 domain scores reported on the 0–100 scale.

Supplementary **Table S3**. Correlations between SF-36 domain scores and the Physical Component Summary (PCS) and Mental Component Summary (MCS) scores.

SF-36 domain	PCS p	p	MCS p	p
Physical Functioning	0.836	<0.001	−0.096	0.055
Role Physical	0.583	<0.001	0.080	0.111
Bodily Pain	0.773	<0.001	0.073	0.143
General Health	0.625	<0.001	0.258	<0.001
Vitality	0.453	<0.001	0.536	<0.001
Social Functioning	0.429	<0.001	0.462	<0.001
Role Emotional	0.320	<0.001	0.373	<0.001
Mental Health	0.226	<0.001	0.773	<0.001

Note: Values are Spearman's rank correlation coefficients (ϱ)

Supplementary **Table S4**. Spearman correlations between the Pittsburgh Sleep Quality Index (PSQI) global score and the SF-36 summary component scores.

Variable	Spearman's ϱ	p-value
PCS	−0.331	<0.001
MCS	−0.351	<0.001
